# *Synotis panzhouensis* (Asteraceae, Senecioneae), a distinct new species with red-purple pappus from southwestern Guizhou, China

**DOI:** 10.3897/phytokeys.166.58654

**Published:** 2020-10-29

**Authors:** Zhi Li, Hai-Lei Zheng, Ming Tang

**Affiliations:** 1 College of Forestry, Guizhou University, Guiyang, 550025, Guizhou, China Guizhou University Guiyang China; 2 Wild Dali Nature Education and Research Center, Dali 671000, Yunnan, China Wild Dali Nature Education and Research Center Dali China; 3 College of Forestry, Jiangxi Agricultural University, Nanchang 330045, Jiangxi, China Jiangxi Agricultural University Nanchang China

**Keywords:** Asteraceae, new species, Senecioneae, taxonomy, *Synotis nayongensis*

## Abstract

A new species of Asteraceae, *Synotis panzhouensis*, is described and illustrated from Guizhou Province in China. Compared with other species of the genus, it is distinguishable by having red-purple pappus; additionally, it differs from its closest ally *S. nayongensis* by the larger involucres and phyllaries, and higher number of phyllaries and disk florets. In addition, detailed discussion of morphological differences, the provisional IUCN status and a distribution map are provided.

## Introduction

*Synotis* (Clarke) C.Jeffrey & Y.L.Chen (Asteraceae, Senecioneae) includes 60 annual and sub-shrubby species predominantly distributed in northern India, Nepal, Bhutan, northern Myanmar, Thailand, Vietnam and southern China (Jeffrey and Chen 1984; Chen 1999; Chen et al. 2011; Tang 2014; Joshi et al. 2013). The genus was separated from *Senecio* L. mainly due to the anther bases having sterile tailed auricles (vs. without sterile tailed auricles) (Jeffrey and Chen 1984). For China, many taxonomic and nomenclatural novelties within the genus have been reported in recent years (Tang et al. 2013a, b, c, 2014, 2017; Tong et al. 2017; Li et al. 2018), and at the present time, approximately 50 species are recorded in China, of which 24 are endemic (Tang 2014, Tang unpublished data).

During an expedition to Panzhou, Guizhou Province, China in 2020, we found an unusual population of *Synotis* at pre-anthesis. Initially, the plants seemed to be similar to *S. nayongensis* C.Jeffrey & Y.L.Chen, but further examination revealed several diagnostic differences between the Guizhou population and *S. nayongensis*; therefore, we describe the Guizhou plants as a new species to science here.

## Material and methods

Herbarium studies were conducted in GZAC, IBSC, JXAU, KUN, and PE. Field observations were made in Panzhou, Guizhou, China in August and September 2020.

## Results and discussion

### Synotis panzhouensis

Taxon classificationPlantaeAsteralesAsteraceae

M.Tang & H.L.Zheng sp. nov.

D6FFFEE5-AE8B-5BC2-8BFD-26C03DE1A670

urn:lsid:ipni.org:names:77212605-1

Figs 1 , 2

#### Type.

China. Guizhou Province, Panzhou, Dazhai village, west of Luotuo valley, limestone mountains, 1803 m a.s.l., herbaceous tier in mixed forest, 26°04'37.59"N, 104°51'47.40"E, 1 September 2020, *Z. Li & M. Tang 2020-0901* (holotype JXAU! isotypes GZAC! JXAU!).

**Figure 1.**
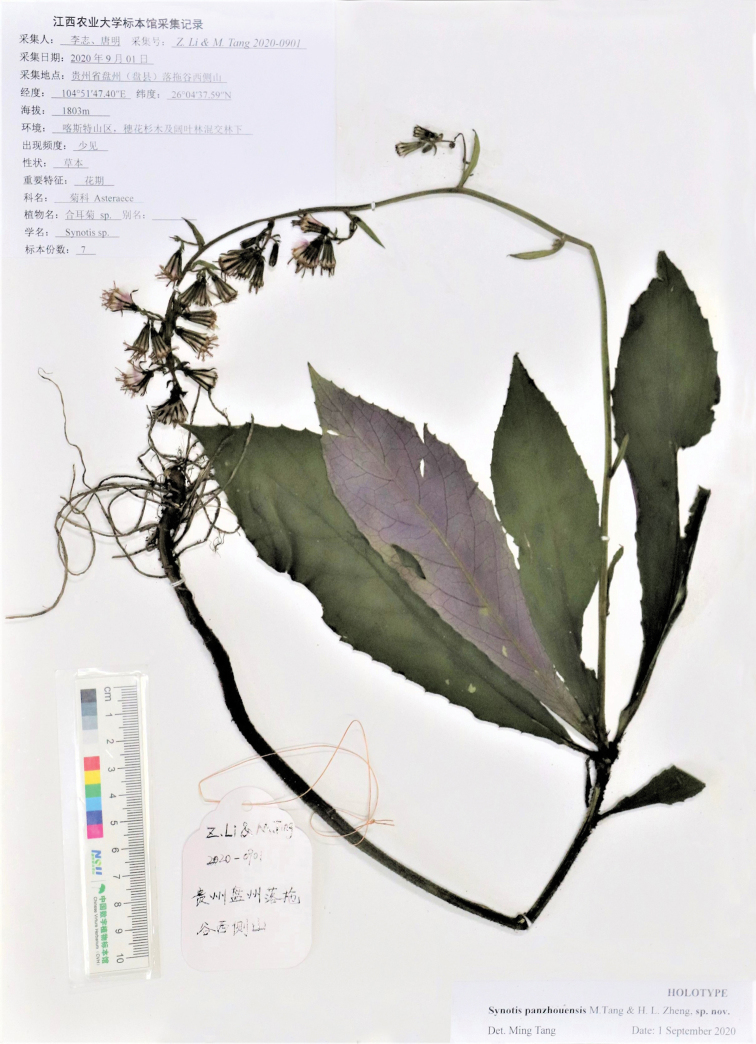
Holotype of *Synotis panzhouensis* (Panzhou, Guizhou, China, *Z. Li & M. Tang 2020-0901* (JXAU)).

#### Diagnosis.

*Synotis panzhouensis* is similar to *S. nayongensis* but differs from the latter species by its larger involucral bracts (7–8 × 3–4 mm vs. 5–6 × 2–2.5 mm) and phyllaries (7–8 × 2–3 mm vs. 5–6 × 1–1.5 mm), higher number of phyllaries ((7) 8 vs. 5) and disk florets (8–11 vs. 4–5), different colour of phyllaries (green vs. white with green apex) and pappus (red-purple vs. white).

**Figure 2.**
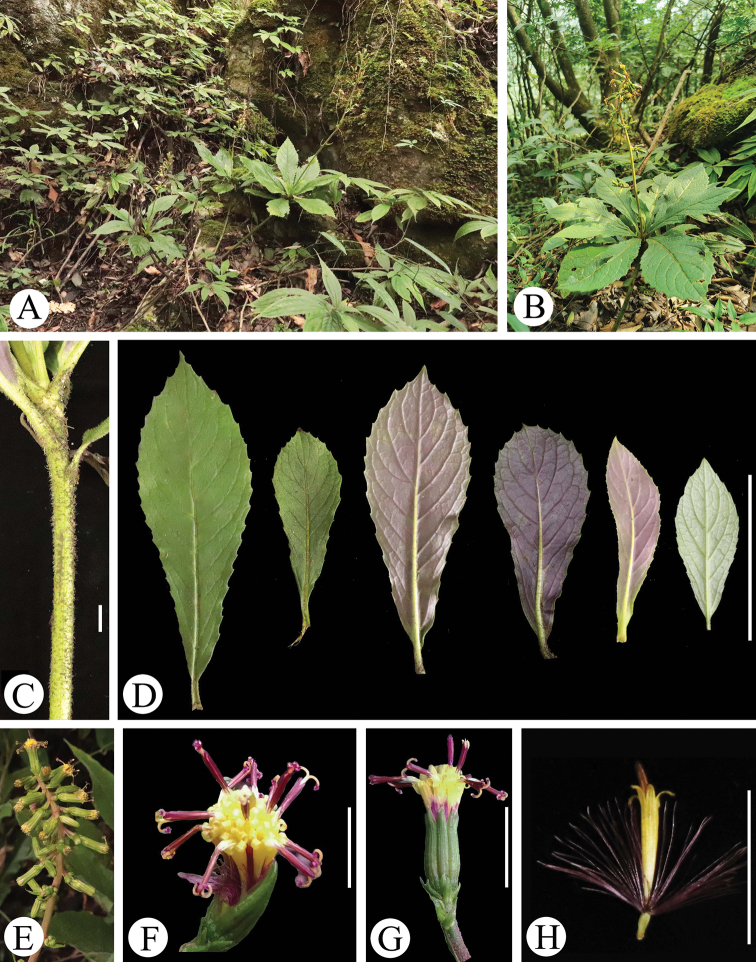
*Synotis panzhouensis* in the wild (Dazhai village, Panzhou, Guizhou, China). **A** habitat **B** general habit **C** portion of vegetative stem **D** leaf blades (left two: adaxial surface; right four: abaxial surface) **E** synflorescence **F** capitulum (top view) **G** capitulum (side view) **H** a disk floret with red-purple pappus. Scale bars: 5 mm (**C**); 10 cm (**D**); 4 mm (**F**); 1 cm (**G, H**).

**Figure 3.**
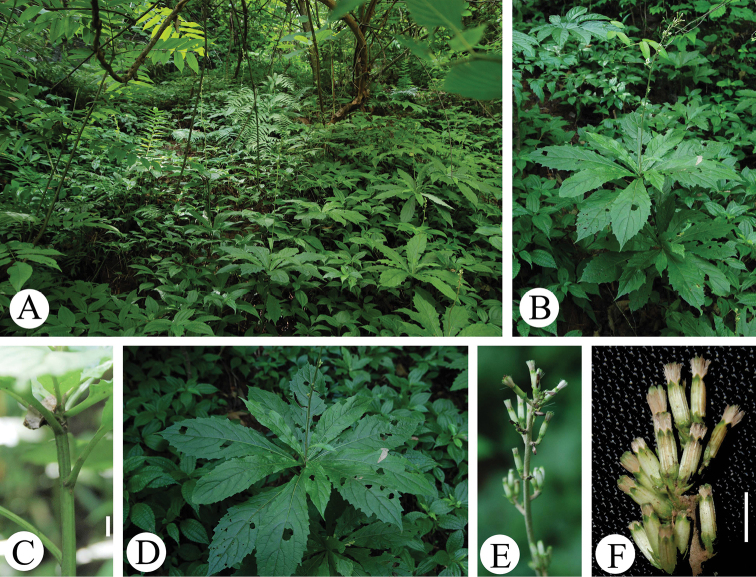
*Synotis nayongensis* in the wild (Luzui village, Nayong, Guizhou,China). **A** habitat **B** habit **C** portion of vegetative stem **D** leaf blades from the adaxil view **E** synflorescence **F** portion of synflorescence (side view, note the white phyllaries with green apex, and also note the white pappus). Scale bars: 5 mm (**C**); 6 mm (**F**).

#### Description.

Perennial herbs, erect, rhizomatous. Rhizome thick, horizontal. Vegetative stem solitary, erect, 20–35 cm, densely white or ferruginous setulose. Flowering stem solitary, erect, scapiform, 30–60 cm tall, shortly branching, fulvous tomentose. Leaves rosulate at the base of fertile shoot; petioles 1–1.5 cm long, not winged, slightly expanded at base; blades oblanceolate or obovate, 10–18 × 2.5–5 cm, papyraceous, abaxially sparsely arachnoid, glabrescent or subglabrous, adaxially scattered setulose, pinnately veined, lateral veins 12–16, arcuate-ascending, base cuneate, margin shallowly sinuate-apiculate or repand-apiculate, apex subacute-acuminate. Stem leaves on reproductive shoots few, narrowly lanceolate, remote, much smaller. Capitula discoid, numerous, arranged in an attenuate narrowly paniculoid thyrse; pedicels 3–5 mm, fulvous tomentose, 1- or 2- bracteate; bracts below capitula linear, 5–10 mm long. Involucres narrowly campanulate, 7–8 × 3–4 mm, calyculate; bracts of calyculus 3–5, ovate-oblong or lanceolate, 1/4–1/3 as long as phyllaries; phyllaries (7) 8, narrowly oblong, 2–3 mm wide, herbaceous, green, margin narrowly scarious, glabrous, not veined, apically triangular, obtuse. Ray florets absent. Disk florets 8–11; corolla yellow, 8–10 mm, with tube 7–8 mm long, limb narrowly funnelform, somewhat exserted from involucre; lobes oblong-lanceolate, 1–2 mm long, apically acute. Anthers 3.5–4 mm long, anther tails equaling anther-collars; appendages lanceolate; anther-collars balusterform, basally dilated. Style branches 1.8–2 mm long, covered with long marginal papillae and often with a central tuft not conspicuously longer. Achenes ca. 2 mm, glabrous. Pappus 8–10 mm long, red-purple.

#### Distribution.

**Figure 4.**
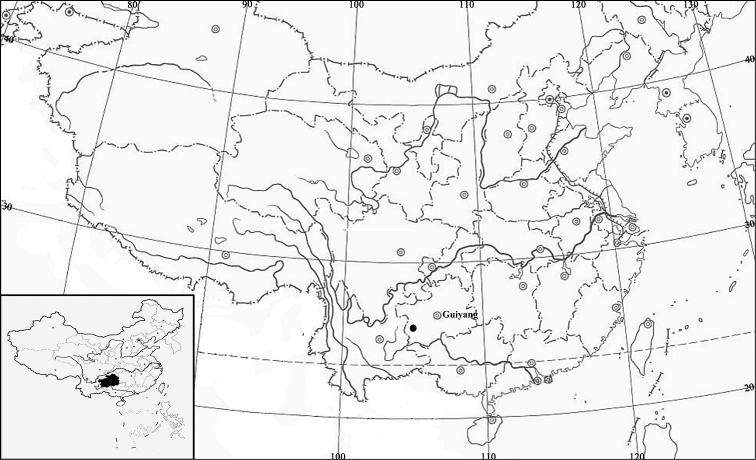
Record of *Synotis panzhouensis*.

*Synotis panzhouensis* is endemic to southwestern Guizhou, China (Fig. 4); to date, only known from the type population.

#### Habitat.

Growing in mixed forest with other herbs in limestone mountains at an elevation of ~1800 m.

#### Phenology.

Flowering from late August to September; fruiting October.

#### Etymology.

The specific epithet is derived from Panzhou, the type locality of the species. The Chinese name is ‘盘州合耳菊 (Pan zhou he er ju)’.

#### Discussion.

As shown in Figs 1–3, *Synotis panzhouensis* is similar to *S. nayongensis* in the oblanceolate or obovate rosulate leaves at the base of synflorescence, the narrow paniculoid thyrse and absence of ray florets. However, we detected significant differences between the two species as listed in Table 1.

*Synotis panzhouensis* is only distributed in Panzhou, southwestern Guizhou, China, while *S. nayongensis* is mainly distributed around northwestern Guizhou (Ruan et al. 2020), with its type locality in Nayong, a county located 200 km NE from Panzhou.

**Table 1.** Comparison of *Synotis panzhouensis* and *S. nayongensis*.

Characters	*Synotis panzhouensis*	*S. nayongensis*
**Indumentum of vegetative stem**	densely white to ferruginous setulose	sparsely pubescent, glabrescent to glabrous
**Petioles**	1–1.5 cm	petiole 1–2.5 cm
**Leaf blades**	adaxially green or purple	adaxially green
**Flowering stem**	fulvous tomentose, not glabrescent	thinly tomentose, glabrescent
**Involucres**	7–8 × 3–4 mm	5–6 × 2–2.5 mm
**Phyllaries**	(7) 8, 7–8 × 2–3 mm, green, not veined	5, 5–6 × 1–1.5 mm, white, apex green, conspicuously 3–5-veined
**Disk florets**	8–11, deep yellow, lamina yellow	4–5, yellowish, lamina yellow-whitish, apex green when young
**Anthers**	red-purple	yellow-whitish
**Achenes**	ca. 2 mm long	ca. 4 mm long
**Pappus**	red-purple	white

From all other species of *Synotis*, *S. panzhouensis* is very distinct in the red-purple pappus. According to our observation of the plants *in vivo*, as well as a critical examination of taxa throughout the genus, most species (~50 spp.) have white pappus. Five species in sect. S. ser. Fulvipapposae C.Jeffrey & Y.L.Chen often have white or stramineous pappus, which is consistent with the descriptions reported by Jeffrey and Chen (1984), Chen (1999) and Chen et al. (2011). The pappus colour of *S. vaniotii* (Lévl.) C.Jeffrey & Y.L.Chen and *S. palmatisecta* Y.L.Chen & D.J.Liu were described as rubescent, but this might be the result of inattentive observation, for both of the species were found with white, or rarely pale brown pappus during our field and herbarium observations. It is noteworthy to mention that *S. chenopodiifolia* (DC.) M.Tang, C.Ren & Q.E.Yang, a species always reported with brown or yellow pappus, has been reported with a dark purple pappus in a population in Gyirong County (Tang et al. 2014), but such variation is rather atypical in the species. *Synotis chenopodiifolia* is a plant to 80–180 cm with triangular-ovate or triangular-hastate leaves and a capitulum with 5 phyllaries, 2 or 3(–4) disk florets, and it obviously differs from the leaf characters and capitulum characters as seen in *S. panzhouensis*.

Morphologically, following the characters and delimitation of Jeffrey and Chen (1984) and Tang (2014), *S. panzhouensis* is best referred to Synotis sect. Synotis ser. Synotis due to the leaves gathered at the apex of the vegetative stem and its terminal inflorescence.

#### IUCN Red List Category.

*Synotis panzhouensis* was found in a single location with an extremely small population in Panzhou, southwestern Guizhou, China. Due to the remote distance and difficult and dangerous accessibility to the type locality, the population was not disturbed by human activity and currently appears to be growing very well. However, according to our observation, the population comprises no more than 100 plants, and with a small geographical range of no more than 1000 m^2^, we recommend that *S. panzhouensis* be categorized as Critically Endangered (CR) using criteria B and C following the IUCN Red List categories (IUCN 2019).

## Supplementary Material

XML Treatment for Synotis panzhouensis
